# Review on Molecular Mechanisms of Antifouling Compounds: An Update since 2012

**DOI:** 10.3390/md15090264

**Published:** 2017-08-28

**Authors:** Lianguo Chen, Pei-Yuan Qian

**Affiliations:** 1 Division of Life Science, Hong Kong University of Science and Technology, Clear Water Bay, Hong Kong, China; lianchen@cityu.edu.hk; 2 State Key Laboratory in Marine Pollution, City University of Hong Kong, Tat Chee Avenue, Kowloon, Hong Kong, China

**Keywords:** antifouling compounds, molecular mechanisms, specific targets, general targets, degradation, toxicity

## Abstract

Better understanding of the mechanisms of antifouling compounds is recognized to be of high value in establishing sensitive biomarkers, allowing the targeted optimization of antifouling compounds and guaranteeing environmental safety. Despite vigorous efforts to find new antifouling compounds, information about the mechanisms of antifouling is still scarce. This review summarizes the progress into understanding the molecular mechanisms underlying antifouling activity since 2012. Non-toxic mechanisms aimed at specific targets, including inhibitors of transmembrane transport, quorum sensing inhibitors, neurotransmission blockers, adhesive production/release inhibitors and enzyme/protein inhibitors, are put forward for natural antifouling products or shelf-stable chemicals. Several molecular targets show good potential for use as biomarkers in future mechanistic screening, such as acetylcholine esterase for neurotransmission, phenoloxidase/tyrosinase for the formation of adhesive plaques, *N*-acyl homoserine lactone for quorum sensing and intracellular Ca^2+^ levels as second messenger. The studies on overall responses to challenges by antifoulants can be categorized as general targets, including protein expression/metabolic activity regulators, oxidative stress inducers, neurotransmission blockers, surface modifiers, biofilm inhibitors, adhesive production/release inhibitors and toxic killing. Given the current situation and the knowledge gaps regarding the development of alternative antifoulants, a basic workflow is proposed that covers the indispensable steps, including preliminary mechanism- or bioassay-guided screening, evaluation of environmental risks, field antifouling performance, clarification of antifouling mechanisms and the establishment of sensitive biomarkers, which are combined to construct a positive feedback loop.

## 1. Introduction

To deter the undesirable colonization of artificial structures by marine organisms, which is referred to as biofouling, it is in common use to coat the submerged surfaces with antifouling paint incorporating biocidal compounds and releasing them at a controlled rate [1,2]. With increasing public awareness and concern for environmental protection, it is generally recognized that the organotin, metals and supplementary booster biocides (e.g., Irgarol 1051, Diuron, copper pyrithione, chlorothalonil, SeaNine 211 and dichlofluanid) constitute a substantial threat to marine ecosystems through environmental pollution and high toxicity [3,4,5]. Therefore, their gradual phase-out is foreseen. As the pressure to find environmentally benign and effective alternatives increases, researchers turn to isolating natural antifouling compounds from a broad array of biological sources (e.g., terrestrial plants, algae, coral, sponge and microbes), as these natural products may possess higher specificity against fouling organisms [6,7]. In addition, improved understanding of settlement cues allows the selection or synthesis of shelf-stable compounds with known modes of action and explores their antifouling potential based on their effects on signaling pathways crucial for settlement (e.g., neurotransmitter signaling, quorum sensing and adhesive production). 

Until now, various compounds with potential antifouling activity have been identified from a library of natural products or shelf-stable chemicals. Although the mechanisms are still poorly understood, current findings document that these promising antifoulants appear to affect settlement through distinct patterns, which can be classified roughly into several categories such as inhibitors of ion channel function, inhibitors of quorum sensing, blockers of neurotransmission or inhibitors of adhesive production or release [5]. Furthermore, for some antifouling compounds, specific target molecules in fouling organisms have been determined, such as blue mussel phenoloxidase, which is necessary for byssus production; acetylcholine esterase (AChE), which is involved in cholinergic neural signaling during the settlement of barnacle cyprids; and N-acyl homoserine lactone (AHL), which mediates quorum sensing during the formation of biofilms. The discovery of these molecular targets has established sensitive biomarkers, facilitating more efficient and accurate screening of antifouling compounds and also highlighting the need to continue to investigate the mechanisms through which antifoulants take effect.

As proposed by the Biocidal Products Regulation (BPR) of the European Union [8], studies to elucidate antifouling mechanisms are regarded as a prerequisite for the registration of any novel antifoulant. A clear understanding of the mode of action of antifouling compounds will help to identify the molecules or pathways that are essential for the settlement of fouling organisms. This understanding will allow the establishment of more sensitive and targeted biomarkers, in turn accelerating preliminary screening. Researchers can also further improve the functional groups of antifouling compounds to improve the coupling with molecular targets, thus achieving more efficient and better targeted antifouling compounds. Therefore, investigation of antifouling mechanisms will certainly not delay the development of antifoulants, but will provide a positive feedback loop through which both will benefit. Since our last review of antifouling mechanisms, new and informative molecular insights have emerged continuously, meriting a summary of new understandings to give an overview of recent progress in antifouling molecular mechanisms. In this review, studies indicating both specific targets and also general responses to the pressure of antifoulants are both included.

## 2. Antifouling Compounds with Proposed Specific Targets

Specific targets are particular molecules or pathways that previous research has proposed to be directly affected by antifouling chemicals to initiate deterrence of settlement. Current knowledge of such specific targets can be divided into five major groups: inhibitors of transmembrane transport, quorum sensing inhibitors, neurotransmission blockers, adhesive production/release inhibitors and enzyme/protein inhibitors (Table 1). 

### 2.1. Inhibitors of Transmembrane Transport

Crude toxin extracted from the Puffer fish *Amblyrhynchotes hypselogenion* and *Lagocephalus sceleratus* demonstrate antifouling activity in the field after incorporation into paints. Tetrodotoxin poisoning is considered responsible for the in-situ antifouling performance, which selectively blocks the sodium channel, inducing paralyzing effects during the generation and transmission of electrical impulses along the peripheral neuromuscular systems [9]. Antifouling compounds also interfere with the homeostasis of cellular calcium ions (Ca^2+^) to inhibit the attachment of fouling organisms. For example, halogenated indole derivatives (i.e., gramine, 6-chloroindole, 7-chloroindole and 6-bromoindole) can trigger the efflux of Ca^2+^ from the intracellular environment and the resulting reduction in Ca^2+^ abundance within cells probably contributes to the inhibition of settlement of fouling organisms (e.g., bacteria and algae) [10,11]. Polyphosphate, a type of orthophosphate polymer, can attach to the bacterial cell membrane and chelate the Ca^2+^ there, resulting in cell death and inhibition of biofilm growth [12]. In addition, transmembrane transport of the amino acid tryptophan is commonly influenced by alkylated guanidinium compounds [13]. Because the biosynthesis of tryptophan is essential for bacterial tolerance to biocides, impaired tryptophan uptake through the membrane is hypothesized to lead to the antibacterial activity of alkylated guanidinium compounds.

### 2.2. Quorum Sensing Inhibitors

The quorum sensing mechanism regulates cell-to-cell communication and plays important roles in the maturation and differentiation of multi-species biofilms. A variety of natural products and shelf-stable compounds inhibit quorum sensing and biofilm development, such as furanosesterterpenes from the sponge *Ircinia felix* [14], 2-dodecanoyloxyethanesulfonate from the red alga *Asparagopsis taxiformis* [15], secochiliolide acid from the Patagonian shrub *Nardophyllum bryoides* [16], diketopiperazines from the microorganisms *Marinobacter* sp. SK-3 and *Rheinheimera japonica* KMM 9513T [19,20], cembranoid diterpenes from the Caribbean gorgonian *Eunicea knighti* [24], and alkyl triphenylphosphonium salts synthesized in the laboratory [25]. Besides, even when immobilized in a coating, acylase can hydrolyze AHL autoinducers through enzymatic activity, thus blocking the transduction of quorum sensing between bacteria cells [26]. In comparison, three isothiocyanate derivatives (i.e., allylisothiocyanate, benzylisothiocyanate and 2-phenylethylisothiocyanate) have the capacity to inhibit quorum sensing by modulating the activity and synthesis of AHL [27]. The mycotoxins patulin and penicillic acid are well-known inhibitors of quorum sensing, whose effect is attributed to inhibition of luxS-encoded autoinducer 2 signaling [28]. The modification of biofilm density and composition by these quorum sensing inhibitors is believed to indirectly affect invertebrate larval attachment. However, it appears that antibacterial activity cannot be directly extrapolated to antifouling performance.

### 2.3. Neurotransmission Blockers

Given the role of AChE in the settlement of invertebrate biofouling organisms, the inhibition of AChE enzymatic activity has been used as a sensitive indicator of antifouling efficacy for diverse compounds, including territrem derivatives from the marine-derived fungus *Aspergillus terreus* SCSGAF0162 [29], pulmonarins A and B from the sub-Arctic ascidian *Synoicum pulmonaria* [30,31], and 3-alkylpyridinium oligomers and polymers (3-APS) as cholinergic antagonists [32]. Inhibition of AChE activity interrupts cholinergic signaling, thereby blocking neurotransmission and reducing the success of settlement of fouling organisms. In addition, the synthetic poly-APS analog APS8 can compete with acetylcholine at the cholinergic receptors, blocking cholinergic neural signals and inducing the hormetic response of barnacle cyprids [33]. Furthermore, histamine neurotransmitter signaling is closely involved in the regulation of the settlement process because histamine receptor antagonists (e.g., triprolidine and cetirizine) can effectively inhibit the attachment and metamorphosis of barnacle cyprids [34,35,36].

### 2.4. Adhesive Production/Release Inhibitors

Because phenoloxidase in blue mussel is a key enzyme involved in both the crosslinking and formation of the adhesive plaques necessary to provide a firm anchor to substrata, inhibition of the activity of this enzyme has frequently been used as a sensitive and efficient biomarker to test antifouling performance. Bromotyrosine derivative ianthelline from the Arctic marine sponge *Stryphnus fortis* [37] and synthetic hemibastadin derivatives [38] strongly inhibit the catalytic activity of blue mussel phenoloxidase, implying their ability to deter settlement of this invertebrate. In addition, synthetic alkyl triphenylphosphonium salts display broad-spectrum antifouling activity against both micro- and macro-fouling together by inhibiting tyrosinase, another model enzyme that is essential for byssus production in mussels [25].

### 2.5. Enzyme/Protein Inhibitors

The red pigment prodigiosin extracted from *Serratia marcescens* CMST 07 is a bacterial secondary metabolite used for antifouling. It is able to pass through the cell membrane and inhibit the DNA-regulating enzymes such as DNA gyrase and topoisomerase IV, inhibiting cell growth [39]. In an ascidian larval bioassay using *Ciona savignyi* Herdman, two allelochemicals (i.e., radicicol and polygodial) strongly inhibit larval metamorphosis with 99% inhibition concentration (IC99) of 0.8 µg/mL and 0.003 µg/mL, respectively [40]. It is speculated that interference between these allelochemicals and heat shock protein (HSP)-90 is responsible for inhibiting the triggering of ascidian metamorphosis. Dibutylphthalate isolated from the marine bacterium *R. japonica* KMM 9513T has antibacterial activity, probably due to glucosidase inhibition that affects energy production for bacterial growth [20]. Nong et al. isolated two natural antifouling products (dicitrinin A and phenol A acid) from the marine gorgonian-derived fungal strain *Xylariaceae* sp. SCSGAF0086 [41]. Dicitrinin A shows enzymatic inhibition of Src homology 2 domain-containing phosphotyrosine phosphatase and inosine monophosphate dehydrogenase, while phenol A acid inhibits cathepsin B. However, the existence of a link between enzyme inhibition and settlement-deterrence of the bryozoan *Bugula neritina* was not clarified for dicitrinin A and phenol A acid [41].

## 3. Antifouling Compounds with Proposed General Targets

General targets are global responses of organisms to the stress of antifouling compounds. These may involve multiple points of attack, without identifying the responsible molecules or pathways directly. Those speculative mechanisms with no direct verification are also included as general targets as a reference to the specific targets. The general targets proposed or conjectured in the literatures have been classified into following categories: Protein expression/metabolic activity regulators, oxidative stress inducers, neurotransmitter blockers, surface modifiers, biofilm inhibitors, adhesive production/release inhibitors and toxic killing (Table 2). 

### 3.1. Protein Expression/Metabolic Activity Regulators 

Exposure of bacterial cells (*Escherichia coli*) to 500 mg/L of the sodium salt of zosteric acid induces alterations in the whole proteomic signature characterized by stress-associated, motility-related, quorum-sensing-associated (LuxS enzyme) and metabolism/biosynthesis-related proteins [42]. It is concluded that bacteria preferentially synthesize various protective proteins, such as quorum sensing and flagella, in response to the challenge of the sodium salt of zosteric acid acting as an environmental cue [42]. Butenolide derived from the deep-sea bacterium *Streptomyces albidoflavus* strain UST040711-291 has been shown to protect coated panels for at least six months in the field after incorporation into soluble matrix paints [43]. Environmental monitoring in natural seawater shows that butenolide degrades quickly with a half-life of 13.0 h [44]. Compared with the commercial booster biocide SeaNine 211, butenolide has relatively lower chronic toxicity towards a marine teleost, the marine medaka *Oryzias melastigma*, in terms of hepatic oxidative stress, AChE inhibition for neurotoxicity, endocrine disruption and reproductive impairment [45]. Proteomic profiling demonstrates that an exposure to low concentrations of butenolide over 28 days primarily disorganizes the cytoskeletal structure in the brains of medaka [46], and activates the detoxification system in their livers to eliminate butenolide quickly from the intracellular environment through bile acid, ensuring lower non-target toxicity and higher biosafety [47]. Poly-ether B, isolated from the sponge-associated bacterium *Winogradskyella poriferorum*, can substantially decrease the cell viability of *Vibrio* sp. 010 [48]. Proteomics research shows that proteins associated with nucleotide metabolism, glyoxylate cycle, and stress responses are mainly altered in expression levels, while metabolomics analysis finds differential changes of metabolites such as tripeptides, fatty acids, and quorum-sensing molecules after treatment with poly-ether B [48]. Biogenic silver nanoparticles produced in the brown algae *Turbinaria ornata* and *T. conoides* display antibacterial and antifouling activities, which are assumed to result from the silver binding with the thiol groups of DNA and RNA to affect protein biosynthesis in bacteria [49,50]. Cochliomycin A from the fungus *Cochliobolus* appreciably affects the protein expression profiles associated with detoxification (cytochrome P450 and glutathione S-transferase) and NO/cGMP pathway in barnacle cyprids [51]. Because NO/cGMP signaling plays a critical role in the settlement of cyprids, agonist and antagonist experiments further indicate that cochliomycin A may exert antifouling activity against barnacles by stimulating this pathway [51]. Moreover, the diterpene (−)14-deoxycrassin, isolated from the soft coral *Sinularia flexibilis*, is also able to reduce the expression of inducible nitric oxide synthase [52]. In addition to protein expression, various metabolic activities appear to be targeted by antifouling compounds, such as cell division and growth by eunicellin-type diterpenoids [53], and energy production by atorvastatin [36] or by synthetic fluorescent probes [55]. Stimulated metabolic activities are known to deplete energy reserves in barnacle cyprids and thus retard settlement through energy deficiency.

### 3.2. Oxidative Stress Inducers

The presence of hexose oxidase in the crude extract of the red seaweed *Chondrus crispus* enzymatically catalyzes the generation of hydrogen peroxide (H_2_O_2_), which oxidatively damages fouling organisms, consequently deterring their attachment [56]. Many antifouling compounds act by the generation of H_2_O_2_, including zinc peroxide (ZnO_2_) [57,58] and zinc oxide nanorod [59]. ZnO or copper nanoparticles in the surface of the coating can photocatalytically produce reactive oxygen species when irradiated by sunlight, resulting in oxidative stress and cell death [60,61]. Chitosan-porphyrin films will also produce reactive oxygen species, mainly singlet oxygen, to selectively kill microorganisms in the presence of light [62]. Some strong oxidizing agents, such as chlorine dioxide and juglone, can attack the thiol groups of biomolecules, interfering with various physiological processes essential for settlement [56,63].

### 3.3. Neurotransmission Blockers

A natural product antifoulant, oleamide from the periostracum of marine mussels (*Mytilus edulis*), can interact with multiple neurotransmitter systems [64]. In addition, the lipid-regulating compound atorvastatin affects the concentration of methyl farnesoate, a potential crustacean hormone [36]. These disturbances to neurotransmission signals by antifouling compounds interrupt the normal attachment and metamorphosis of fouling organisms.

### 3.4. Surface Modifiers

Antifouling compounds, such as polygodial from the canelo tree *Drimys winteri* [65,66], synthetic 3-alkylpyridinium oligomers and polymers (3-APS) [32] and linoleic acid from the semi-evergreen plant *Dryopteris crassirhizoma* [67], show surfactant properties known to disrupt or solubilize the cell membrane of fouling organisms. In addition, cationic antifouling chemicals can interact with negatively-charged bacterial cells to cause lysis of cell membranes and leakage of cellular contents [57,58,60,68,69,70]. The lipophilic nature of thymol and eugenol enables their interaction with the lipid bilayer of cellular membranes, allowing membrane insertion and altering the fluidity and permeability of cell membranes [71]. Another strategy of surface modification employed by antifouling compounds is to either increase the roughness and hydrophilicity of substrata [57,58,72] or decrease the hydrophobicity of bacterial surfaces [73], thus reducing the strength of attachment of fouling organisms. 

### 3.5. Biofilm Inhibitors

The antibacterial activity of 7-hydroxy-4-methylcoumarin can be attributed to the inhibition of bacterial nucleic acid synthesis and quorum sensing by its coumarin ring, which reduces the formation of biofilms on surfaces [74]. Modified black wattle tannin has the capacity to chelate metals in solution, especially iron [75]. Because metals are essential for the growth of microorganisms, the removal of metals by tannic acid tends to deter bacteria. Besides, the binding of silver ions or nanoparticles to sulfur or phosphorus containing biomolecules will probably cause cell death of bacteria during the development of biofilms [76].

### 3.6. Adhesive Production/Release Inhibitors

The so-called “living paint” demonstrates antibacterial and antifouling activities by immobilizing the marine bacteria *Pseudomonas aeruginosa* 1242 directly in the coating [77]. Changes in the composition of biofilms and amylase proteolytic activity on adhesives are two probable effects whose interaction contributes to the antifouling performance of “living paint”. Synthetic poly(l-lactic acid) releases lactic acid slowly, and the resulting acidity may inhibit crosslinking reactions and the formation of networks of cement proteins in barnacle cyprids, thus decreasing the settlement percentage [78]. 

### 3.7. Toxic Killing

The natural product 3,3′-diindolylmethane is isolated from the *Pseudovibrio denitrificans* UST4-50 and exhibits potent antifouling activity against the settlement of barnacles and bryozoans in paint formulations, whose field performance is comparable with the commercial antifouling agent SeaNine 211 [79]. However, environmental risk assessments consistently verify that 3,3′-diindolylmethane is too stable to biodegrade quickly in the marine environment and also has potent endocrine disrupting effects towards non-target organisms. For example, after 28-days of chronic exposure of marine medaka to 8.5 µg/L of 3,3′-diindolylmethane, sex-specific responses emerged, that is, estrogenic effects in males but anti-estrogenic effects in females along the entire hypothalamus–pituitary–gonadal-liver (HPGL) axis [80]. The lower estradiol/testosterone ratio reduces the synthesis of vitellogenin and eggshell proteins, consequently blocking the development and maturation of oocytes in the ovary [81]. Reproductive failure is shown by reduced fecundity and the obviously decreased viability of offspring larvae after parental exposure to 3,3′-diindolylmethane. Therefore, although 3,3′-diindolylmethane originates as a natural product, not all natural products are readily degradable and environmentally benign. Systematic evaluation of their environmental risks is indispensable. 

Although organotin has been banned for use as an antifouling additive, pollution from it is still distributed worldwide due to its persistent property and continuous use. Organotin induces resistance in microalgae by pre-selective mutations [82]. In the abalone *Haliotis diversicolor*, 28-days of exposure to organotin compounds not only disturbs both energy production and osmotic balance but also induces oxidative stress [83]. Reduced reproductive success caused by organotin is also observed in the ascidian *C. intestinalis*, partly through alterations in the electrical property of the oocyte plasma membrane [84]. Suppression of the immune function is also found in another ascidian, *Botryllus schlosseri* [85]. In the wood frog (*Lithobates sylvaticus*), organotin disrupts lipid metabolism and signaling transduction via the retinoid-X-receptor and perixosomal proliferation receptor gamma in both acute and chronic exposure regimes [86]. As pigments in antifouling coating, heavy metals, including copper and cadmium, can increase larval abnormalities significantly and induce DNA damage in the pacific oyster (*Crassostrea gigas*) [88].

To resist algal accumulation on immersed structures, diverse booster biocides, previously used as herbicides, fungicides or bactericides, are used as supplements in copper-based paint formulations. However, the use of these antifouling booster biocides does not go through comprehensive evaluation of their environmental risks. Following large-scale use in antifouling paints, their environmental pollution and high non-target toxicity pose non-negligible threats to marine ecosystems. Irgarol 1051 is adopted from herbicides and is known to inhibit the photosynthesis and carbon incorporation of the sugar kelp *Saccharina latissima* [89], and retard the cell cycle of the marine green alga *Ostreococcus tauri* [90]. In addition, growth of the freshwater cyanobacterium *Synechococcus* sp. PCC7942 is stimulated by Irgarol 1051, with altered physiological composition of both soluble proteins and polysaccharide content in addition to the induction of oxidative stress [91]. As compensation for inhibited photosynthesis, Irgarol 1051 exerts a selection bias for tolerant algal species in the marine periphyton communities [92]. Further, exposure to Italic 1051 increases the abnormality and DNA strand breaks in the larvae of Pacific oyster (*C. gigas*) [88]. In vitro treatment shows that cell apoptosis through mitochondrial dysfunction and oxidative stresses is induced by Irgarol 1051 [93].

Similarly, inhibited photosynthesis and carbon incorporation are detected for SeaNine 211 in the sugar kelp *S. latissima* [89]. SeaNine 211 exposure induces oxidative stress by dramatically depleting the intracellular pools of glutathione [92], or changing antioxidant enzyme activity [94]. A 28-day exposure to 1.0 µg/L of SeaNine 211 also increases apoptosis in testicular germ cells of the marine teleost mummichog *Fundulus heteroclitus*, probably through a caspase-dependent pathway [95]. Furthermore, in the marine medaka *O. melastigma*, chronic exposure to environmentally observed concentrations of SeaNine 211 for 28 days induces hepatic oxidative stress [45], decreases AChE activity and disrupts the mitogen-activated protein kinase (MAPK) signaling pathway in brain tissues [45,46] and causes endocrine disruptive effects through the entire HPGL axis [47,96]. Imbalanced hormonal homeostasis and increased estradiol/testosterone ratio indicate an estrogenic intracellular environment. Transgenerational impairment is also characterized by the delayed hatching and lethargic swimming of offspring larvae. To directly identify the binding target by which subsequent secondary effects are mediated, a pull-down assay has been developed with SeaNine 211 immobilized on the surface of agarose beads. The interaction of proteins with the functional isothiazolinone group shows that SeaNine 211 has a high binding affinity to G protein alpha subunits in the brains of two teleosts (i.e., marine medaka and zebrafish) and can competitively inhibit signal transduction through G protein-coupled receptors, which may result in the consequent endocrine disruption [97].

Diuron is capable of inhibiting the photosynthesis, carbon incorporation and progression through the cell cycle in macroalgae [89,90]. The depressed photosynthetic performance and the resultant physiochemical changes decrease the sinking rate of marine diatoms, which probably alters their survival strategy [98]. In addition, the abnormal sodium currents and conductance of the oocyte plasma membrane caused by Diuron may impair the reproductive fitness and population sustainability of the ascidian *C. intestinalis* [84]. Copper pyrithione can affect the settlement and growth of marine periphyton communities by disrupting the integrity of cell membrane as a consequence of increased cellular metal ion concentrations [92]. A series of adverse effects, including the altered composition of the periphyton community [99], suppression of the immune function [85] and the induction of oxidative stress [100] have been documented for zinc pyrithione. Similarly, dichlofluanid, tolyfluanid and chlorothalonil can inhibit the photosynthesis and carbon incorporation of the sugar kelp *S. atissima* [89]. Further, tolyfluanid disrupts folate synthesis and inhibits thiol-containing enzymes by forming disulfide bridges during the settlement and growth of marine periphyton communities [92]. In mature oocytes of the ascidian *C. intestinalis*, a decrease in the amplitudes of sodium and fertilization currents by chlorothalonil is observed, suggesting an involvement of plasma membrane ion currents in the teratogenic mechanism of chlorothalonil [101].

## 4. Conclusions

This review gives a summary of progress in the understanding of antifouling mechanisms since 2012, providing a timely supplement to our last review of these mechanisms [5]. Briefly, literature that investigates or speculates on the molecular mechanisms of antifoulants is included, and categorized as either specific targets or general targets, based on whether or not certain target molecules and pathways are involved. Specific targets embrace five groups, including inhibitors of transmembrane transport, quorum sensing inhibitors, neurotransmission blockers, adhesive production/release inhibitors and enzyme/protein inhibitors (Table 1). General targets roughly comprise six groups: protein expression or metabolic activity regulators, oxidative stress inducers, neurotransmission blockers, surface modifiers, biofilm inhibitors, adhesive production or release inhibitors and toxic killing (Table 2). Comparison with the overall complicated responses of general targets that give a blurry assumption, direct identification or speculation about the specific intracellular targets of antifouling compounds is certainly of great value in discovering the mechanisms responsible for antifouling activity more clearly. In the specific target groups, target molecules such as AChE, phenoloxidase and tyrosinase, AHL of quorum sensing, intracellular Ca^2+^ levels and HSP-90 have the potential to be developed and employed as sensitive and efficient biomarkers. Another advantage of these proposed biomarkers is that their detection methods are generally available, which can be directly and easily adopted from previous studies for mechanistic antifouling screening in future research.

It is necessary to point out that, although a large variety of natural products or shelf-stable compounds have been tested for antifouling activity, there is currently little information for their modes of action. This situation has not improved even slightly since our last review [5]. It is well recognized that increased knowledge of antifouling mechanisms will in turn facilitate more targeted and efficient screening of antifouling compounds. Therefore, future work on antifouling must combine both chemical and biological work to provide a more complete picture of antifouling performance. 

**Figure 1:**
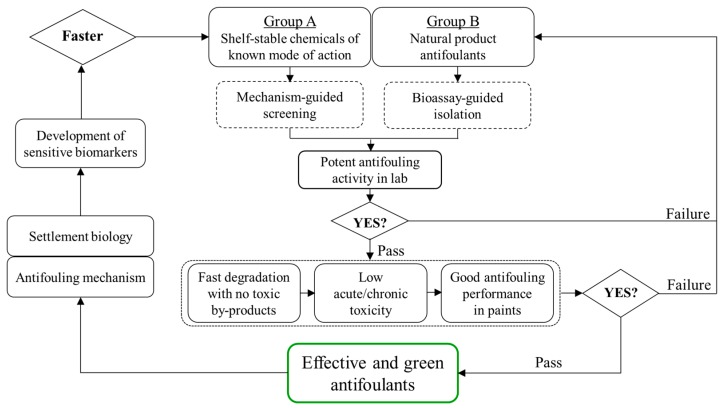
Proposed workflow for the development of novel antifouling compounds either extracted from the library of shelf-stable chemicals of known mode of action or isolated from biological samples as natural product antifoulants. Mechanism- or bioassay-guided screening is employed for shelf-stable chemicals or natural products, respectively, to establish a database of antifoulants with potent activity. Then, further verification based on three aspects (i.e., environmental fate, biosafety and antifouling in coatings) is included to ascertain that the antifoulants not only deter settlement effectively but also are environmentally “green”. In turn, research on antifouling mechanism and settlement molecular insight will facilitate the utility of sensitive biomarkers for faster screening of promising antifouling compounds.

In view of the present status and knowledge gaps regarding the development of antifoulants, we propose here a workflow to list the issues that need to be resolved step by step prior to the commercialization of any novel antifouling chemicals (Figure 1). First, shelf-stable or natural product compounds are purchased, synthesized or isolated for preliminary screening based on the mechanism-guided or bioassay-guided method, respectively. Those compounds showing potent antifouling activities in laboratory conditions will be further evaluated for: (1) their degradation kinetics in various environmental matrices to ensure that these promising antifoulants degrade quickly once released from coatings but without generating more toxic byproducts, thus maximally avoiding possible environmental pollution; (2) their environmental biosafety in both acute and chronic exposure scenarios to ensure that these promising antifoulants will have low adverse effects against non-target marine organisms of different trophic levels; and (3) their field antifouling performance after incorporation into paints to ensure that these promising antifoulants are compatible with paint formulations and demonstrate efficacious and durable performance in the field as well as in the laboratory. These three aspects are expected to support each other systematically for any effective and environmentally friendly antifouling compound. Next, studies to give insights into both the mechanisms of antifouling activities and the biological clues during settlement will assist the establishment of sensitive and targeted biomarkers, facilitating faster screening of antifouling alternatives in turn.

## Figures and Tables

**Table 1:** Molecular mechanisms and bioactivity of antifouling compounds with proposed specific targets.

Proposed Molecular Mechanism and Targets	Compounds	Activity	Category	Sources	Toxicity	References
**Inhibitors of Transmembrane Transport**						
Blocking selectively the sodium channel to paralyze the peripheral neuromuscular system	Crude toxin extracts	Antifouling in paint	Natural product	Puffer fish *Amblyrhynchotes hypselogenion* and *Lagocephalus sceleratus*	Toxic	[9]
Triggering algal cellular Ca^2+^ efflux	Gramine, 6-chloroindole, 7-chloroindole, 6-bromoindole	Antibacterial and anti-algae	Shelf-stable	Halogenated indole derivatives	Non-toxic	[10,11]
Removing Ca^2+^ from the cell membrane and causing cell death	Polyphosphate	Antibacterial	Shelf-stable	Orthophosphate polymer	Non-toxic	[12]
Affecting tryptophan amino acid import through membrane	Alkylated guanidinium compounds	Antimicrobial (yeast *Saccharomyces cerevisae*)	Shelf-stable	Synthetic in lab	Non-toxic	[13]
**Quorum Sensing Inhibitors**						
Inhibiting quorum sensing	Furanosesterterpenes	Antibacterial	Natural product	Spong *Ircinia felix*	Non-toxic	[14]
Quorum sensing inhibition	2-Dodecanoyloxyethanesulfonate	Antibacterial	Natural product	Red alga *Asparagopsis taxiformis*	Non-toxic	[15]
Inhibiting biofilm formation through interference with quorum sensing	Secochiliolide acid	Antifouling (diatom, algae, bryozoan, tubeworm, ascidian)	Natural product	Patagonian shrub *Nardophyllum bryoides*	Non-toxic	[16]
Quorum sensing inhibition	Crude extract	Antibacterial; antifouling (diatom, bryozoan *Bugula neritina*)	Natural product	Invasive brown macroalga *Sargassum muticum*	Non-toxic	[17]
Inhibiting quorum sensing	Crude extract	Antibacterial	Natural product	Macroalgae from the Brazilian coast	Non-toxic	[18]
Bacteiral quorum-sensing inhibitory activity	Diketopiperazines	Antibacterial	Natural product	Microorganism *Marinobacter* sp. SK-3 and *Rheinheimera japonica* KMM 9513T	Non-toxic	[19,20]
Potent quorum-sensing attenuation to inhibit the growth of biofilms	A low molecular mass compound	Antibacterial	Natural product	Coral-associated bacterial isolates	Non-toxic	[21]
Quorum-sensing inhibitory activity	A combination of fungal secondary metabolites and fatty acids	Antibacterial	Natural product	Marine endophytic fungal isolates from coral *Diploria strigosa*	Non-toxic	[22]
Quorum-sensing inhibition	Ethanol extracts	Antibacterial	Natural product	Gorgonian corals *Pseudopterogorgia americana*, *P. acerosa*, and *Pseudoplexuara flexuosa*	Non-toxic	[23]
Quorum-sensing inhibition and biofilm inhibition	Cembranoid diterpenes	Antibacterial	Natural product	Caribbean gorgonian *Eunicea knighti*	Non-toxic	[24]
Non-toxic quorum sensing disruptors	Alkyl triphenylphosphonium Salts	Antimicrobial (marine bacteria, fungi, diatom); Antifouling (macroalgae *Gayralia oxysperma*, mussel *Mytilus galloprovincialis*)	Shelf-stable	Synthetic in lab	Non-toxic	[25]
Hydrolysis of N-acyl homoserine lactone (AHL) autoinducers	Acylase	Antibacterial	Shelf-stable	Enzymes	Non-toxic	[26]
Quorum sensing inhibition by modulating AHL activity and synthesis	Allylisothiocyanate, benzylisothiocyanate and 2-phenylethylisothiocyanate	Antibacterial	Shelf-stable	Isothiocyanates	Non-toxic	[27]
Inhibitory effect on luxS-encoded autoinducer 2 signaling	Patulin and penicillic acid	Antibacterial	Shelf-stable	Mycotoxin	Toxic	[28]
**Neurotransmission Blockers**						
Strong inhibitor of acetylcholine esterase (AChE)	Territrem derivatives	Antifouling (*Balanus amphitrite*)	Natural product	Marine-derived fungus *Aspergillus terreus* SCSGAF0162	Non-toxic	[29]
Reversible and noncompetitive AChE inhibitors	Pulmonarins A and B	Antibacterial	Natural product	Sub-Arctic ascidian *Synoicum pulmonaria*	Non-toxic	[30,31]
Interruption of cholinergic system through AChE inhibition	3-Alkylpyridinium oligomers and polymers (3-APS)	Antimicrobial (bacteria, fungi); antifouling	Shelf-stable	Cholinergic antagonist	Non-toxic	[32]
Competition with acetylcholine to receptors and inhibition of the cholinergic system	Poly-APS analog APS8	Antifouling (*B. amphitrite*)	Shelf-stable	Synthetic in lab	Non-toxic	[33]
Influencing histamine neurotransmitter signaling for photoreceptors	Triprolidine and cetirizine	Antifouling (*B. amphitrite*)	Shelf-stable	Histamine receptor antagonist	Non-toxic	[34,35,36]
**Adhesive Production/Release Inhibitors**						
Strong inhibitors of blue mussel phenoloxidase	Bromotyrosine derivative ianthelline	Antibacterial; antifouling (microalgae, barnacle *B. improvisus*, blue mussel *M. edulis*)	Natural product	Arctic marine sponge *Stryphnus fortis*	Non-toxic	[37]
Potent inhibitors of blue mussel phenoloxidase	Hemibastadin derivatives	Antifouling (blue mussel *M. edulis*)	Shelf-stable	Synthetic in lab	Non-toxic	[38]
Inhibitory activity on tyrosinase for mussel byssal production	Alkyl triphenylphosphonium salts	Antimicrobial (marine bacteria, fungi, diatom); Antifouling (macroalgae *G. oxysperma*, mussel *M. galloprovincialis*)	Shelf-stable	Synthetic in lab	Non-toxic	[25]
**Enzyme/Protein Inhibitors**						
Inhibiting target DNA modulating enzymes to block bacterial growth	Red pigment prodigiosin	Antibacterial; antifouling (cyanobacteria *Synechococcus* sp.; *B. amphitrite*)	Natural product	*Serratia marcescens* CMST 07	Non-toxic	[39]
Interference with HSP-90 to inhibit metamorphosis	Radicicol and polygodial	Antifouling (ascidian *Ciona savignyi*)	Shelf-stable	Allelochemicals	Non-toxic	[40]
Glucosidase inhibition to affect energy production	Dibutylphthalate	Antibacterial	Natural product	Marine bacterium *R. japonica* KMM 9513T	Non-toxic	[20]
Enzymatic inhibitory activities towards Src homology 2 domain-containing phosphotyrosine phosphatase and inosine monophosphate dehydrogenase	Dicitrinin A	Antifouling (*B. neritina*)	Natural product	Marine gorgonian-derived fungal strain *Xylariaceae* sp. SCSGAF0086	Non-toxic	[41]
Inhibitory activity towards cathepsin B	Phenol A acid	Antifouling (*B. neritina*)	Natural product	Marine gorgonian-derived fungal strain *Xylariaceae* sp. SCSGAF0086	Non-toxic	[41]

**Table 2:** Molecular mechanisms and bioactivity of antifouling compounds with proposed general targets.

Proposed Molecular Mechanism and Targets	Compounds	Activity	Category	Sources	Toxicity	References
**Protein Expression/Metabolic Activity Regulators**						
Leading to global stress on cells and favoring the expression of quorum-sensing and flagella synthesis	Zosteric acid sodium salt	Antibacterial	Shelf-stable	Synthetic in lab	Non-toxic	[42]
Initiating detoxifying systems to ensure fast elimination from organisms and lower non-specific toxicity	Butenolide	Antibacterial; antifouling (barnacle *Balanus amphitrite*; tubeworm *Hydroides elegans*; bryozoan *Bugula neritina*)	Natural product	*Streptomyces albidoflavus* strain UST040711-291	Non-toxic	[43,44,45,46,47]
Affecting protein expression related to nucleotide metabolism, the glyoxylate cycle, and stress responses	Poly-ether B	Antibacterial	Natural product	Sponge-associated bacterium *Winogradskyella poriferorum*	Non-toxic	[48]
Binding with thiol groups of DNA and RNA and affect the protein biosynthesis of bacteria	Biogenic silver nanoparticles	Antibacterial; antifouling (barnacle *B. amphitrite*)	Natural product	Brown alga *Turbinaria ornata* and *T. conoides*	Non-toxic	[49,50]
Affected the cytochrome P450, glutathione S-transferase (GST) and NO/cGMP pathways	Cochliomycin A	Antifouling (barnacle *B. amphitrite*)	Natural product	Fungus *Cochliobolus*	Non-toxic	[51]
Reducing the expression of inducible nitric oxide synthase (iNOS) and cyclooxygenase 2 (COX-2)	Diterpenes: (−)14-deoxycrassin	Antifouling (bryozoan *B. neritina*, barnacle *B. albicostatus*)	Natural product	Soft coral *Sinularia flexibilis*	Non-toxic	[52]
Inhibitory activities of cell division and growth	Eunicellin-type diterpenoids	Antifouling (barnacle *B. amphitrite*)	Natural product	Chinese gorgonian *Astrogorgia* sp.	Non-toxic	[53]
Increasing metabolic activity, depleting energy reserve of cyprids and retarding settlement	Atrovastatin	Antifouling (barnacle *B. amphitrite*)	Shelf-stable	Lipid-regulating compound	Non-toxic	[36]
Lowering pH values and releasing sorbic acid into the cytoplasm to inhibit many metabolic functions	Ferric sorbate and aluminum sorbate	Antifouling in paint (diatom, seaweed, barnacle, tubeworm, bryozoan, ascidian)	Shelf-stable	Synthetic in lab	Non-toxic	[54]
Acting in the oil cell region and attaching to the carapace surface to induce agglutination of cyprids	Fluorescent probes	Antifouling (barnacle *B. amphitrite*)	Shelf-stable	Synthetic in lab	Non-toxic	[55]
Able to inhibit RNA transcription	Usnic acid	Antibacterial	Shelf-stable	Dibenzofuran derivative	Non-toxic	[56]
**Oxidative Stress Inducers**						
Enzymatic generation of hydrogen peroxide (H_2_O_2_) by hexose oxidase	Crude extract	Antibacterial	Natural product	Red seaweed *Chondrus crispus*	Non-toxic	[56]
Reacting with seawater to create H_2_O_2_	Zinc peroxide (ZnO_2_)	Antibacterial and antifouling	Shelf-stable	Strong oxidizing agent	Non-toxic	[57,58]
Production of H_2_O_2_ on the surface of the coating	Zinc oxide nanorod (ZnO)	Antibacterial; antifouling (algae, barnacle) in field	Shelf-stable	Synthetic in lab	Non-toxic	[59]
Photocatalytic generation of reactive oxygen species by ZnO nanoparticles	Chitosan/ZnO nanocomposite	Antimicrobial (bacteria, fungi, microalgae)	Shelf-stable	Synthetic in lab	Non-toxic	[60]
Formation of reactive oxygen species resulting in cell death	Chitosan-decorated copper nanoparticles	Antibacterial	Shelf-stable	Synthetic in lab	Non-toxic	[61]
Producing reactive oxygen species to selectively kill microorganisms	Chitosan-porphyrin films	Antibacterial (*Listeria innocua*)	Shelf-stable	Synthetic in lab	Non-toxic	[62]
Attacking the sulfhydryl groups of biomolecules	Chlorine dioxide	Antibacterial; antifouling (barnacle *B. reticulatus*)	Shelf-stable	Potent oxidant	Toxic	[63]
Interfering with vital cell processes	Juglone	Antibacterial	Shelf-stable	Potent oxidant	Non-toxic	[56]
**Neurotransmission Blockers**						
Interacting with multiple neurotransmitter systems	Oleamide	Antifouling (algae *Porphyra suborbiculata*)	Natural product	Marine mussels (*Mytilus edulis*)	Non-toxic	[64]
Affecting the concentration of methyl farnesoate, a potential crustacean hormone	Atrovastatin	Antifouling (barnacle *B. amphitrite*)	Shelf-stable	Lipid-regulating compound	Non-toxic	[36]
**Surface Modifiers**						
Nonionic surfactant properties to disrupt the cell membrane	Polygodial	Antibacterial; antifouling (microalgae, Ascidian *Ciona savignyi*, barnacle *B. improvisus*, mussel, tubeworm)	Natural product	Canelo tree *Drimys winteri*	Non-toxic	[65,66]
Surfactant and lysis of cell membrane and microbes	3-Alkylpyridinium oligomers and polymers (3-APS)	Antimicrobial (bacteria, fungi); antifouling	Shelf-stable	Synthetic in lab	Non-toxic	[32]
Detergent properties at high concentrations to solubilize the membrane	Linoleic acid	Antibacterial	Natural product	Semi-evergreen plant *Dryopteris crassirhizoma*	Non-toxic	[67]
Interacting with bacterial membrane to allow for membrane insertion	Cationic micropeptides	Antibacterial; antifouling (algae, barnacle *B. improvisus*)	Shelf-stable	Synthetic in lab	Non-toxic	[68]
Selective lysis of microbial membranes and subsequent killing of bacteria	Natural resin acid-derived cationic compounds and polymers	Antibacterial	Shelf-stable	Synthetic in lab	Non-toxic	[69]
Interacting with the negative charges of the microbial cell membrane due to cationic nature of chitosan	Chitosan/ZnO nanocomposite	Antimicrobial (bacteria, fungi, microalgae)	Shelf-stable	Synthetic in lab	Non-toxic	[60]
Interacting and decomposing the negatively-charged cell membrane	Polyhexamethylene guanidine molybdate	Antibacterial; antifouling (bryozoan, Dreissenidae mollusk)	Shelf-stable	Synthetic in lab	Non-toxic	[70]
Cationic binding to negatively charged bacterial cell walls	Chlorhexidine	Antibacterial and antifouling	Shelf-stable	Cationic molecule	Non-toxic	[57,58]
Interacting with the lipid bilayer of cytoplasmic membranes and causing loss of integrity	Thymol and eugenol	Antifouling (barnacle, tubeworm, bryozoan, ascidian, algae)	Shelf-stable	Lipophilic phenolic compounds	Non-toxic	[71]
Altering the roughness of surfaces and the contacts of cyprid antennular discs	Nano-sized carbon black	Antifouling (barnacle *B. amphitrite*)	Shelf-stable	Carbon-based nanomaterials	Non-toxic	[72]
Increasing hydrophilic surface and thereby reducing the adhesion of microorganisms	Tween 85	Antibacterial and antifouling	Shelf-stable	Non-ionic surfactant	Non-toxic	[57,58]
Affecting the EPS production, growth and the surface hydrophobicity of the biofilm-forming bacteria	Coconut husk extract (phenolic compounds)	Antibacterial	Natural product	Coconut *Cocos nucifera* L.	Non-toxic	[73]
**Biofilm Inhibitors**						
Inhibition of bacterial nucleic acid synthesis and reduce biofilm formation via quorum sensing inhibition	7-Hydroxy-4-methylcoumarin	Antibacterial; antifouling (diatom, algae, bryozoan, tubeworm, ascidian, mussel)	Shelf-stable	Synthetic in lab	Non-toxic	[74]
Removing metals essential for the growth of microorganisms	Modified black wattle tannin	Antibacterial; Antifouling in field	Shelf-stable	Chemically modified in lab	Non-toxic	[75]
Binding to sulfur and phosphorus containing biomolecules and causing cell damage	Poly ethylene glycol based silver nanocomposites	Antibacterial	Shelf-stable	Synthetic in lab	Non-toxic	[76]
**Adhesive Production/Release Inhibitors**						
Proteolytic and amylase enzyme activity on the adhesives of settling organisms	Bacterial immobilization in paint (“living paint”)	Antibacterial; antifouling (diatom, polychaete, bryozoan, algae)	Natural product	Marine strain *Pseudomonas aeruginosa* 1242	Non-toxic	[77]
Inhibiting cross-linking reactions of cement proteins due to acidity	Poly(l-lactic acid)	Antifouling (barnacle *B. amphitrite*) in lab and field	Shelf-stable	Synthetic in lab	Non-toxic	[78]
**Toxic Killing**						
Strong endocrine disruptor	3,3′-Diindolylmethane	Antifouling (barnacle *B. Amphitrite*, bryozoan *B. neritina*)	Natural product	*Pseudovibrio denitrificans* UST4-50	Toxic	[79,80,81]
Disturbing energy metabolism and osmotic balance; induce oxidative stress; immunosuppression; reproductive impairment; disrupting signaling transduction	Organotin	Antifouling	Heavy metal	Organometallics	Toxic	[82,83,84,85,86,87]
Increasing larval abnormalities and DNA damage	Copper; cadmium	Antifouling	Heavy metal		Toxic	[88]
Inhibiting the photosynthesis; genotoxic; oxidative stress; inhibiting cell cycle and inducing apoptosis	Irgarol 1051	Antifouling	Booster biocide	Herbicide	Toxic	[88,89,90,91,92,93]
Inhibiting the photosynthesis; oxidative stress; endocrine disruption and reproductive impairment	Sea-Nine 211	Antifouling	Booster biocide	Isothiazolone compound	Toxic	[45,46,47,89,92,94,95,96,97]
Inhibiting the photosynthesis; oxidative stress; inhibiting cell cycle and hatching; reproductive impairment	Diuron	Antifouling	Booster biocide	Herbicide	Toxic	[84,89,90,91,98]
Disrupting the cell membrane through apoptosis	Copper pyrithione	Antialgae	Booster biocide	Fungicide	Toxic	[92]
Changing the composition of the periphyton community; immunosuppressive toxicity; oxidative stress	Zinc pyrithione	Antifouling	Booster biocide	Bactericide; fungicide; algicide	Toxic	[85,99,100]
Inhibition of photosynthesis and carbon incorporation	Dichlofluanid	Antialgae	Booster biocide	Fungicide	Toxic	[89]
Inhibition of photosynthesis and carbon incorporation; disrupting folate synthesis and inhibiting thiol-containing enzymes	Tolyfluanid	Antialgae	Booster biocide	Fungicide	Toxic	[89,92]
Inhibiting the photosynthesis; reproductive impairment and teratogenic	Chlorothalonil	Antifouling	Booster biocide	Fungicide	Toxic	[89,101]

## References

[1] Yebra D.M., Kiil S., Dam-Johansen K. (2004). Antifouling technology-past, present and future steps towards efficient and environmentally friendly antifouling coatings. Prog. Org. Coat..

[2] Callow J.A., Callow M.E. (2011). Trends in the development of environmentally friendly fouling-resistant marine coatings. Nat. Commun..

[3] Omae I. (2003). Organotin antifouling paints and their alternatives. Appl. Organomet. Chem..

[4] Voulvoulis N. (2006). Antifouling paint booster biocides: Occurrence and partitioning in water and sediments. Handb. Environ. Chem..

[5] Qian P.Y., Chen L., Xu Y. (2013). Mini-review: Molecular mechanisms of antifouling compounds. Biofouling.

[6] Fusetani N. (2004). Biofouling and antifouling. Nat. Prod. Rep..

[7] Qian P.Y., Li Z., Xu Y., Li Y., Fusetani N. (2015). Mini-review: Marine natural products and their synthetic analogs as antifouling compounds: 2009–2014. Biofouling.

[8] (2012). Regulation (EU) No 528/2012 of the European Parliament and of the Council of 22 May 2012 Concerning the Making Available on the Market and Use of Biocidal Products.

[9] Soliman Y.A., Mohamed A.S., NaserGomaa M. (2014). Antifouling activity of crude extracts isolated from two Red Sea puffer fishes. Egypt. J. Aquat. Res..

[10] Yang C.Y., Yu Y.N., Sun W.J., Xia C.H. (2014). Indole derivatives inhibited the formation of bacterial biofilm and modulated Ca^2+^ efflux in diatom. Mar. Pollut. Bull..

[11] Yang C., Sun W., Liu S., Xia C. (2015). Comparative effects of indole derivatives as antifouling agents on the growth of two marine diatom species. Chem. Ecol..

[12] Müller W.E.G., Wang X., Guo Y.W., Schröder H.C. (2012). Potentiation of the cytotoxic activity of copper by polyphosphate on biofilm-producing bacteria: A bioinspired approach. Mar. Drugs.

[13] Bowie D., Parvizi P., Duncan D., Nelson C.J., Fyles T.M. (2013). Chemical-genetic identification of the biochemical targets of polyalkyl guanidinium biocides. Org. Biomol. Chem..

[14] Quintana J., Brango-Vanegas J., Costa G.M., Castellanos L., Arévalo C., Duque C. (2015). Marine organisms as source of extracts to disrupt bacterial communication: Bioguided isolation and identification of quorum sensing inhibitors from *Ircinia felix*. Rev. Bras. Farmacogn..

[15] Jha B., Kavita K., Westphal J., Hartmann A., Schmitt-Kopplin P. (2013). Quorum sensing inhibition by *Asparagopsis taxiformis*, a marine macro alga: Separation of the compound that interrupts bacterial communication. Mar. Drugs.

[16] Pérez M., García M., Sánchez M., Stupak M., Mazzuca M., Palermo J.A., Blustein G. (2014). Effect of secochiliolide acid isolated from the Patagonian shrub *Nardophyllum bryoides* as active component in antifouling paints. Int. Biodeter. Biodegrad..

[17] Schwartz N., Dobretsov S., Rohde S., Schupp P.J. (2017). Comparison of antifouling properties of native and invasive *Sargassum* (Fucales, Phaeophyceae) species. Eur. J. Phycol..

[18] Batista D., Carvalho A., Costa R., Coutinho R., Dobretsov S. (2014). Extracts of macroalgae from the Brazilian coast inhibit bacterial quorum sensing. Bot. Mar..

[19] Abed R.M.M., Dobretsov S., Al-Fori M., Gunasekera S.P., Sudesh K., Paul V.J. (2013). Quorum-sensing inhibitory compounds from extremophilic microorganisms isolated from a hypersaline cyanobacterial mat. J. Ind. Microbiol. Biotechnol..

[20] Kalinovskaya N.I., Romanenko L.A., Kalinovsky A.I. (2017). Antibacterial low-molecular-weight compounds produced by the marine bacterium *Rheinheimera japonica* KMM 9513T. Anton. Van Leeuwenhoek.

[21] Golberg K., Pavlov V., Marks R.S., Kushmaro A. (2013). Coral-associated bacteria, quorum sensing disrupters, and the regulation of biofouling. Biofouling.

[22] Martín-Rodríguez A.J., Reyes F., Martín J., Pérez-Yépez J., León-Barrios M., Couttolenc A., Espinoza C., Trigos A., Martín V.S., Norte M. (2014). Inhibition of bacterial quorum sensing by extracts from aquatic fungi: First report from marine endophytes. Mar. Drugs.

[23] Hunt L.R., Smith S.M., Downum K.R., Mydlarz L.D. (2012). Microbial regulation in gorgonian corals. Mar. Drugs.

[24] Tello E., Castellanos L., Arévalo-Ferro C., Duque C. (2012). Disruption in quorum-sensing systems and bacterial biofilm inhibition by cembranoid diterpenes isolated from the octocoral *Eunicea knighti*. J. Nat. Prod..

[25] Martín-Rodríguez A.J., Babarro J.M.F., Lahoz F., Sansón M., Martín V.S., Norte M., Fernández J.J. (2015). From broad-spectrum biocides to quorum sensing disruptors and mussel repellents: Antifouling profile of alkyl triphenylphosphonium salts. PLoS ONE.

[26] Lee J., Lee I., Nam J., Hwang D.S., Yeon K.M., Kim J. (2017). Immobilization and stabilization of acylase on carboxylated polyaniline nanofibers for highly effective antifouling application via quorum quenching. ACS Appl. Mater. Interfaces.

[27] Borges A., Serra S., Abreu A.C., Saavedra M.J., Salgado A., Simões M. (2014). Evaluation of the effects of selected phytochemicals on quorum sensing inhibition and in vitro cytotoxicity. Biofouling.

[28] Liaqat I., Bachmann R.T., Edyvean R.G.J. (2014). Type 2 quorum sensing monitoring, inhibition and biofilm formation in marine microrganisms. Curr. Microbiol..

[29] Nong X.H., Wang Y.F., Zhang X.Y., Zhou M.P., Xu X.Y., Qi S.H. (2014). Territrem and butyrolactone derivatives from a marine-derived fungus *Aspergillus terreus*. Mar. Drugs.

[30] Tadesse M., Svenson J., Sepcic K., Trembleau L., Engqvist M., Andersen J.H., Jaspars M., Stensvåg K., Haug T. (2014). Isolation and synthesis of pulmonarins A and B, acetylcholinesterase inhibitors from the colonial ascidian *Synoicum pulmonaria*. J. Nat. Prod..

[31] Trepos R., Cervin G., Hellio C., Pavia H., Stensen W., Stensvåg K., Svendsen J.-S., Haug T., Svenson J. (2014). Antifouling compounds from the sub-arctic ascidian *Synoicum pulmonaria*: Synoxazolidinones A and C, pulmonarins A and B, and synthetic analogues. J. Nat. Prod..

[32] Grandič M., Zovko A., Frangež R., Turk T., Sepčić K. (2012). Binding and permeabilization of lipid bilayers by natural and synthetic 3-alkylpyridinium polymers. Bioorg. Med. Chem..

[33] Piazza V., Dragić I., Sepčić K., Faimali M., Garaventa F., Turk T., Berne S. (2014). Antifouling activity of synthetic alkylpyridinium polymers using the barnacle model. Mar. Drugs.

[34] Jin C., Qiu J., Miao L., Feng K., Zhou X. (2014). Antifouling activities of anti-histamine compounds against the barnacle *Amphibalanus* (= *Balanus*) *amphitrite*. J. Exp. Mar. Biol. Ecol..

[35] Jin C., Qiu J., Yu S., Miao L., Zhou X. (2014). Histamine promotes the larval metamorphic competence of barnacle *Amphibalanus amphitrite*. Mar. Biol. Res..

[36] Al-Aidaroos A.M., Satheesh S., Devassy R.P. (2017). Effects of pharmacological compounds on the barnacle larval development, metabolism and settlement. Int. Biodeter. Biodegrad..

[37] Hanssen K.Ø., Cervin G., Trepos R., Petitbois J., Haug T., Hansen E., Andersen J.H., Pavia H., Hellio C., Svenson J. (2014). The bromotyrosine derivative ianthelline isolated from the arctic marine sponge *Stryphnus fortis* inhibits marine micro- and macrobiofouling. Mar. Biotechnol..

[38] Niemann H., Hagenow J., Chung M.Y., Hellio C., Weber H., Proksch P. (2015). SAR of sponge-inspired hemibastadin congeners inhibiting blue mussel phenoloxidase. Mar. Drugs.

[39] Ananda Priya K., Satheesh B., Ashok Kumar P., Varalakshmi G., Sivakumar N. (2013). Antifouling activity of prodigiosin from estuarine isolate of *Serratia marcescens* CMST 07. Microbiol. Res. Agro-Ecosyst. Manag..

[40] Cahill P., Heasman K., Jeffs A., Kuhajek J., Mountfort D. (2012). Preventing ascidian fouling in aquaculture: Screening selected allelochemicals for anti-metamorphic properties in ascidian larvae. Biofouling.

[41] Nong X.H., Zheng Z.H., Zhang X.Y., Lu X.H., Qi S.H. (2013). Polyketides from a marine-derived fungus *Xylariaceae* sp.. Mar. Drugs.

[42] Villa F., Remelli W., Forlani F., Vitali A., Cappitelli F. (2012). Altered expression level of *Escherichia coli* proteins in response to treatment with the antifouling agent zosteric acid sodium salt. Environ. Microbiol..

[43] Chen L., Xia C., Qian P.Y. (2017). Optimization of antifouling coatings incorporating butenolide, a potent antifouling agent via field and laboratory tests. Prog. Org. Coat..

[44] Chen L., Xu Y., Wang W.X., Qian P.Y. (2015). Degradation kinetics of a potent antifouling agent, butenolide, under various environmental conditions. Chemosphere.

[45] Chen L., Ye R., Xu Y., Gao Z., Au D.W.T., Qian P.Y. (2014). Comparative safety of the antifouling compound butenolide and 4,5-dichloro-2-n-octyl-4-isothiazolin-3-one (DCOIT) to the marine medaka (*Oryzias melastigma*). Aquat. Toxicol..

[46] Chen L., Zhang H., Sun J., Wong Y.H., Han Z., Au D.W.T., Bajic V.B., Qian P.Y. (2014). Proteomic changes in brain tissues of marine medaka (*Oryzias melastigma*) after chronic exposure to two antifouling compounds: Butenolide and 4,5-dichloro-2-n-octyl-4-isothiazolin-3-one (DCOIT). Aquat. Toxicol..

[47] Chen L., Sun J., Zhang H., Au D.W.T., Lam P.K.S., Zhang W., Bajic V.B., Qiu J.W., Qian P.Y. (2015). Hepatic proteomic responses in marine medaka (*Oryzias melastigma*) chronically exposed to antifouling compound butenolide [5-octylfuran-2(5H)-one] or 4,5-dichloro-2-n-octyl-4-isothiazolin-3-one (DCOIT). Environ. Sci. Technol..

[48] Chandramouli K.H., Dash S., Zhang Y., Ravasi T., Qian P.Y. (2013). Proteomic and metabolomic profiles of marine *Vibrio* sp. 010 in response to an antifoulant challenge. Biofouling.

[49] Vijayan S.R., Santhiyagu P., Singamuthu M., Ahila N.K., Jayaraman R., Ethiraj K. (2014). Synthesis and characterization of silver and gold nanoparticles using aqueous extract of seaweed, *Turbinaria conoides*, and their antimicrofouling activity. Sci. World J..

[50] Krishnan M., Sivanandham V., Hans-Uwe D., Murugaiah S.G., Seeni P., Gopalan S., Rathinam A.J. (2015). Antifouling assessments on biogenic nanoparticles: A field study from polluted offshore platform. Mar. Pollut. Bull..

[51] Wang K., Zhang G., Sun J., Xu Y., Han Z., Liu L., Shao C., Liu Q., Wang C., Qian P.Y. (2016). Cochliomycin A inhibits the larval settlement of *Amphibalanus amphitrite* by activating the NO/cGMP pathway. Biofouling.

[52] Wang J., Su P., Gu Q., Li W.D., Guo J.L., Qiao W., Feng D.Q., Tang S.A. (2017). Antifouling activity against bryozoan and barnacle by cembrane diterpenes from the soft coral *Sinularia flexibilis*. Int. Biodeter. Biodegrad..

[53] Lai D., Liu D., Deng Z., van Ofwegen L., Proksch P., Lin W. (2012). Antifouling eunicellin-type diterpenoids from the gorgonian *Astrogorgia* sp.. J. Nat. Prod..

[54] Pérez M., García M., Stupak M., Blustein G. (2014). Synthesis and characterization of ferric sorbate and aluminum sorbate as antifouling pigments for marine paints. Ind. Eng. Chem. Res..

[55] Fujiwara S., Akima C., Nogata Y., Yoshimura E., Chiba K., Kitano Y. (2013). Bio-organic and anti-barnacle studies of fluorescence-labeled probe compounds against cyprids of barnacles. J. Exp. Mar. Biol. Ecol..

[56] Salta M., Wharton J.A., Dennington S.P., Stoodley P., Stokes K.R. (2013). Anti-biofilm performance of three natural products against initial bacterial attachment. Int. J. Mol. Sci..

[57] Faÿ F., Carteau D., Linossier I., Delbury M., Vallée-Réhel K. (2013). Joint-action of antifouling substances in copper-free paints. Colloid Surf. B.

[58] Carteau D., Vallée-Réhel K., Linossier I., Quiniou F., Davy R., Compère C., Delbury M., Faÿ F. (2014). Development of environmentally friendly antifouling paints using biodegradable polymer and lower toxic substances. Prog. Org. Coat..

[59] Mostafaei A., Nasirpouri F. (2013). Preparation and characterization of a novel conducting nanocomposite blended with epoxy coating for antifouling and antibacterial applications. J. Coat. Technol. Res..

[60] Al-Naamani L., Dobretsov S., Dutta J., Burgess J.G. (2017). Chitosan-zinc oxide nanocomposite coatings for the prevention of marine biofouling. Chemosphere.

[61] Abiraman T., Balasubramanian S. (2017). Synthesis and characterization of large-scale (<2 nm) chitosan-decorated copper nanoparticles and their application in antifouling coating. Ind. Eng. Chem. Res..

[62] Castro K.A.D.F., Moura N.M.M., Fernandes A., Faustino M.A.F., Simões M.M.Q., Cavaleiro J.A.S., Nakagaki S., Almeida A., Cunha Â. (2017). Control of *Listeria innocua* biofilms by biocompatible photodynamic antifouling chitosan based materials. Dyes Pigm..

[63] Venkatnarayanan S., Murthy P.S., Kirubagaran R., Venugopalan V.P. (2017). Chlorine dioxide as an alternative antifouling biocide for cooling water systems: Toxicity to larval barnacle *Amphibalanus reticulatus* (Utinomi). Mar. Pollut. Bull..

[64] Kang J.Y., Bangoura I., Cho J.Y., Joo J., Choi Y.S., Hwang D.S., Hong Y.K. (2016). Antifouling effects of the periostracum on algal spore settlement in the mussel *Mytilus edulis*. Fish. Aquat. Sci..

[65] Cahill P.L., Heasman K., Jeffs A., Kuhajek J. (2013). Laboratory assessment of the antifouling potential of a soluble-matrix paint laced with the natural compound polygodial. Biofouling.

[66] Moodie L.W.K., Trepos R., Cervin G., Larsen L., Larsen D.S., Pavia H., Hellio C., Cahill P., Svenson J. (2017). Probing the structure–activity relationship of the natural antifouling agent polygodial against both micro- and macrofoulers by semisynthetic modification. J. Nat. Prod..

[67] Jung J.E., Pandit S., Jeon J.G. (2014). Identification of linoleic acid, a main component of the n-hexane fraction from *Dryopteris crassirhizoma*, as an anti-*Streptococcus mutans* biofilm agent. Biofouling.

[68] Trepos R., Cervin G., Pile C., Pavia H., Hellio C., Svenson J. (2015). Evaluation of cationic micropeptides derived from the innate immune system as inhibitors of marine biofouling. Biofouling.

[69] Wang J., Chen Y.P., Yao K., Wilbon P.A., Zhang W., Ren L., Zhou J., Nagarkatti M., Wang C., Chu F., He X., Decho A.W., Tang C. (2012). Robust antimicrobial compounds and polymers derived from natural resin acids. Chem. Commun..

[70] Protasov A., Bardeau J.-F., Morozovskaya I., Boretska M., Cherniavska T., Petrus L., Tarasyuk O., Metelytsia L., Kopernyk I., Kalashnikova L., Dzhuzha O., Rogalsky S. (2017). New promising antifouling agent based on polymeric biocide polyhexamethylene guanidine molybdate. Environ. Toxicol. Chem..

[71] Pérez M., García M., Blustein G. (2015). Evaluation of low copper content antifouling paints containing natural phenolic compounds as bioactive additives. Mar. Environ. Res..

[72] Mesarič T., Sepčič K., Piazza V., Gambardella C., Garaventa F., Drobne D., Faimali M. (2013). Effects of nano carbon black and single-layer graphene oxide on settlement, survival and swimming behaviour of *Amphibalanus amphitrite* larvae. Chem. Ecol..

[73] Viju N., Satheesh S., Vincent S.G.P. (2013). Antibiofilm activity of coconut (*Cocos nucifera* Linn.) husk fibre extract. Saudi J. Biol. Sci..

[74] Pérez M., García M., Ruiz D., Autino J.C., Romanelli G., Blustein G. (2016). Antifouling activity of green-synthesized 7-hydroxy-4-methylcoumarin. Mar. Environ. Res..

[75] Peres R.S., Armelin E., Alemán C., Ferreira C.A. (2015). Modified tannin extracted from black wattle tree as an environmentally friendly antifouling pigment. Ind. Crops Prod..

[76] Punitha N., Saravanan P., Mohan R., Ramesh P.S. (2017). Antifouling activities of β-cyclodextrin stabilized peg based silver nanocomposites. Appl. Surf. Sci..

[77] Kharchenko U., Beleneva I., Dmitrieva E. (2012). Antifouling potential of a marine strain, *Pseudomonas aeruginosa* 1242, isolated from brass microfouling in Vietnam. Int. Biodeter. Biodegrad..

[78] Ishimaru N., Tsukegi T., Wakisaka M., Shirai Y., Nishida H. (2012). Effects of poly(l-lactic acid) hydrolysis on attachment of barnacle cypris larvae. Polym. Degrad. Stab..

[79] Wang K., Xu Y., Lu L., Li Y., Han Z., Zhang J., Shao C., Wang C., Qian P.Y. (2015). Low-toxicity diindol-3-ylmethanes as potent antifouling compounds. Mar. Biotechnol..

[80] Chen L., Ye R., Zhang W., Hu C., Zhou B., Peterson D.R., Au D.W.T., Lam P.K.S., Qian P.Y. (2016). Endocrine disruption throughout the hypothalamus-pituitary-gonadal-liver (HPGL) axis in marine medaka (*Oryzias melastigma*) chronically exposed to 3,3′-diindolylmethane (DIM). Chem. Res. Toxicol..

[81] Chen L., Au D.W.T., Hu C., Zhang W., Zhou B., Cai L., Giesy J.P., Qian P.Y. (2017). Linking genomic responses of gonads with reproductive impairment in marine medaka (*Oryzias melastigma*) exposed chronically to the chemopreventive and antifouling agent, 3,3′-diindolylmethane (DIM). Aquat. Toxicol..

[82] Costas E., Gonzalez R., Lo’pez-Rodas V., Huertas I.E. (2013). Mutation of microalgae from antifouling sensitivity to antifouling resistance allows phytoplankton dispersal through ships’ biofouling. Biol. Invasions.

[83] Lu J., Feng J., Cai S., Chen Z. (2017). Metabolomic responses of *Haliotis diversicolor* to organotin compounds. Chemosphere.

[84] Gallo A., Tosti E. (2013). Adverse effect of antifouling compounds on the reproductive mechanisms of the ascidian *Ciona intestinalis*. Mar. Drugs.

[85] Cima F., Ballarin L. (2015). Immunotoxicity in ascidians: Antifouling compounds alternative to organotins—IV. The case of zinc pyrithione. Comp. Biochem. Phys. C.

[86] Higley E., Tompsett A.R., Giesy J.P., Hecker M., Wiseman S. (2013). Effects of triphenyltin on growth and development of the wood frog (*Lithobates sylvaticus*). Aquat. Toxicol..

[87] Iyapparaj P., Revathi P., Ramasubburayan R., Prakash S., Anantharaman P., Immanuel G., Palavesam A. (2013). Antifouling activity of the methanolic extract of *Syringodium isoetifolium*, and its toxicity relative to tributyltin on the ovarian development of brown mussel *Perna indica*. Ecotoxicol. Environ. Saf..

[88] Mai H., Cachot J., Brune J., Geffard O., Belles A., Budzinski H., Morin B. (2012). Embryotoxic and genotoxic effects of heavy metals and pesticides on early life stages of pacific oyster (*Crassostrea gigas*). Mar. Pollut. Bull..

[89] Johansson P., Eriksson K.M., Axelsson L., Blanck H. (2012). Effects of seven antifouling compounds on photosynthesis and inorganic carbon use in sugar kelp *Saccharina latissima* (Linnaeus). Arch. Environ. Contam. Toxicol..

[90] Sanchez-Ferandin S., Leroy F., Bouget F.Y., Joux F. (2013). A new, sensitive marine microalgal recombinant biosensor using luminescence monitoring for toxicity testing of antifouling biocides. Appl. Environ. Microbiol..

[91] Deng X., Gao K., Sun J. (2012). Physiological and biochemical responses of *Synechococcus* sp. PCC7942 to Irgarol 1051 and diuron. Aquat. Toxicol..

[92] Arrhenius Å., Backhaus T., Hilvarsson A., Wendt I., Zgrundo A., Blanck H. (2014). A novel bioassay for evaluating the efficacy of biocides to inhibit settling and early establishment of marine biofilms. Mar. Pollut. Bull..

[93] Wang L., Liang B., Li L., Liu W. (2013). Induction of HepG2 cell apoptosis by Irgarol 1051 through mitochondrial dysfunction and oxidative stresses. Toxicol. In Vitro.

[94] Cima F., Ferrari G., Ferreira N.G.C., Rocha R.J.M., Serôdio J., Loureiro S., Calado R. (2013). Preliminary evaluation of the toxic effects of the antifouling biocide Sea-Nine 211™ in the soft coral *Sarcophyton* cf. *glaucum* (Octocorallia, Alcyonacea) based on PAM fluorometry and biomarkers. Mar. Environ. Res..

[95] Ito M., Mochida K., Ito K., Onduka T., Fujii K. (2013). Induction of apoptosis in testis of the marine teleost mummichog *Fundulus heteroclitus* after in vivo exposure to the antifouling biocide 4,5-dichloro-2-n-octyl-3(2H)-isothiazolone (Sea-Nine 211). Chemosphere.

[96] Chen L., Zhang W., Ye R., Hu C., Wang Q., Seemann F., Au D.W.T., Zhou B., Giesy J.P., Qian P.Y. (2016). Chronic exposure of marine medaka (*Oryzias melastigma*) to 4,5-dichloro-2-n-octyl-4-isothiazolin-3-one (DCOIT) reveals its mechanism of action in endocrine disruption via the hypothalamus-pituitary-gonadal-liver (HPGL) axis. Environ. Sci. Technol..

[97] Chen L., Au D.W.T., Hu C., Peterson D.R., Zhou B., Qian P.Y. (2017). Identification of molecular targets for 4,5-dichloro-2-n-octyl-4-isothiazolin-3-one (DCOIT) in teleosts: New insight into mechanism of toxicity. Environ. Sci. Technol..

[98] Khanam M.R.M., Shimasaki Y., Hosain M.Z., Mukai K., Tsuyama M., Qiu X., Tasmin R., Goto H., Oshima Y. (2017). Diuron causes sinking retardation and physiochemical alteration in marine diatoms *Thalassiosira pseudonana* and *Skeletonema marinoi-dohrnii* complex. Chemosphere.

[99] Ohlauson C., Blanck H. (2014). A comparison of toxicant-induced succession for five antifouling compounds on marine periphyton in SWIFT microcosms. Biofouling.

[100] Avelelas F., Martins R., Oliveira T., Maia F., Malheiro E., Soares A.M.V.M., Loureiro S., Tedim J. (2017). Efficacy and ecotoxicity of novel anti-fouling nanomaterials in target and non-target marine species. Mar. Biotechnol..

[101] Gallo A., Tosti E. (2015). Reprotoxicity of the antifoulant chlorothalonil in ascidians: An ecological risk assessment. PLoS ONE.

